# Closure of a Bronchoesophageal Fistula After Lung Transplantation With an Amplatzer Occluder Device

**DOI:** 10.1016/j.atssr.2022.12.001

**Published:** 2022-12-10

**Authors:** Erik J. Orozco-Hernandez, David McGiffin, Gregory Von Mering, Mustafa Ahmed, Joseph Thachuthara-George, Kondal R. Kyanam-Kabir-Baig, Charles W. Hoopes

**Affiliations:** 1 Division of Cardiothoracic Surgery, Department of Surgery, University of Alabama at Birmingham, Birmingham, Alabama; 2 Division of Cardiothoracic Surgery, Department of Surgery, Alfred Hospital and Monash University, Melbourne, Australia; 3 Division of Cardiovascular Disease, Department of Medicine, HCA Florida Heart and Lung, Ocala, Florida; 4 Division of Cardiovascular Disease, Department of Medicine, University of Alabama at Birmingham, Birmingham, Alabama; 5 Division of Pulmonary, Allergy, and Critical Care Medicine, University of Alabama at Birmingham, Birmingham, Alabama; 6 Department of Medicine, University of Alabama at Birmingham, Birmingham, Alabama

## Abstract

Lung transplantation is associated with a higher morbidity and mortality compared with transplantation of other solid organs, which is related, in part, to the occurrence of airway complications. A bronchoesophageal fistula associated with dehiscence of the right bronchial anastomosis developed in a 59-year-old man after bilateral lung transplantation. Multiple endoscopic and bronchoscopic stent procedures failed to close the fistula. Five months after the lung transplant, the fistula was successfully closed with an Amplatzer ADO septal occluder device. One year later, the fistula was still closed, but because of the development of multiple gastrointestinal complications, the patient received palliative care.

Airway fistula after lung transplantation is uncommon but can be a challenging complication to manage. Patients experience increased mortality and morbidity and decreased quality of life. Surgery is an important treatment option but is inadvisable for some patients. Interventional bronchoscopic procedures, such as stent placement, have low likelihood of success and can be associated with lethal complications. We report a case of a bronchoesophageal fistula associated with dehiscence of the right bronchial anastomosis after bilateral lung transplantation (BLT). After multiple failed endoscopic and bronchoscopic stent procedures, an Amplatzer device was used to successfully close the fistula.

A 59-year-old white man underwent BLT for pulmonary fibrosis. He was readmitted 1 week later with a right-sided empyema, and esophagography revealed an esophageal perforation thought to be caused by the erosion of a hemoclip placed in a lymph node. He underwent placement of an esophageal 23 × 120-mm EndoMaxx stent (Merit), and a chest tube was placed for drainage. Five days later, bronchoscopy demonstrated dehiscence of the right bronchial anastomosis communicating with the mediastinum. A self-expanding bronchial uncovered metal stent (Ultraflex; Boston Scientific) was placed. A right-sided empyema again developed; a chest tube was placed, and esophagography revealed a bronchoesophageal fistula. During the next 5 months, he underwent multiple esophageal stent procedures. Several endoscopic procedures failed to seal the fistula, including over-the-scope clip and tissue BioGlue (CryoLife) using a rigid bronchoscope. Finally, the patient underwent successful closure of the bronchoesophageal fistula with an Amplatzer ADO septal occluder device (Abbott).

The procedure was performed by simultaneous bronchoscopy and esophageal endoscopy. The fistula track (Figure 1A) was noted along the anastomosis site and in the middle third of the esophagus. A stiff angled Glidewire (Terumo) was passed through the bronchoscope and through the fistula into the stomach. A 4F Navicross catheter (Terumo) was loaded inside of the 5F delivery catheter. Both were advanced and crossed the defect. The Navicross catheter was then removed. An Amplatzer ADO II 6 × 6 × 12-mm device was then inserted; the delivery catheter was retracted just distal to the defect. The distal disk was then exposed and visualized in the esophagus. The device was retracted to the defect and then fully deployed, visualizing the proximal disk in the bronchus.

**Figure 1:**
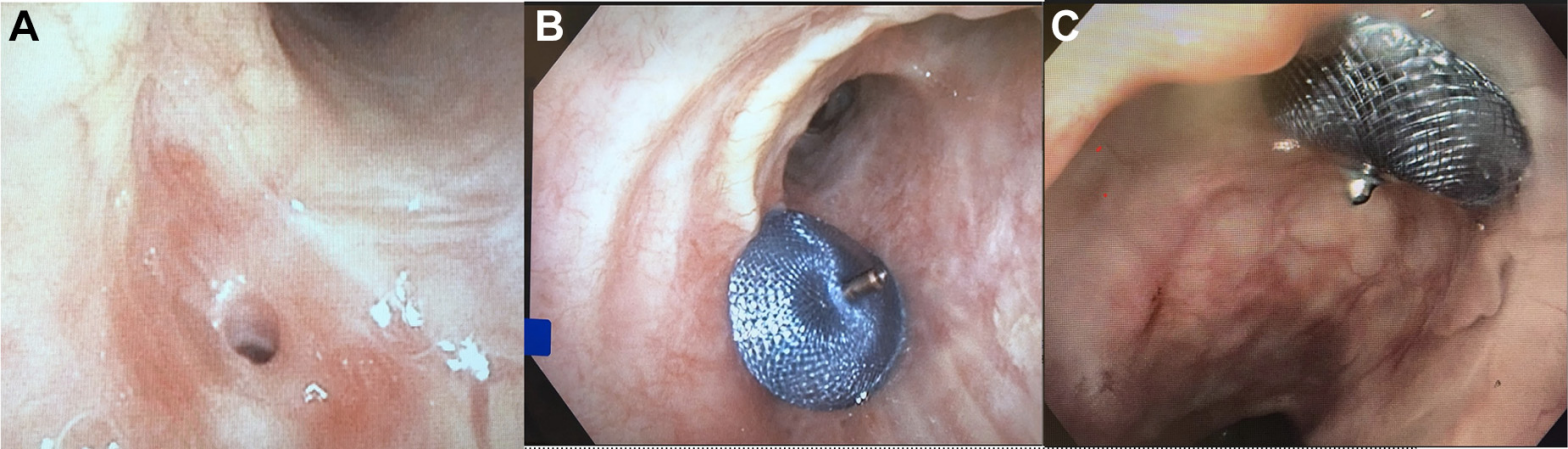
(A) Bronchial side of the fistula before cardiac septal occluder placement. (B) Device from the esophageal examination. (C) Device from airway endoscope examination.

The device was thought to be well seated on both sides (Figures 1B, 1C). There was no obstruction. A tug test was performed, demonstrating adequate anchoring. Intraluminal contrast material was injected through the esophageal endoscope and confirmed an adequate seal with no leakage of contrast material (Figure 2). The device was then released.

**Figure 2:**
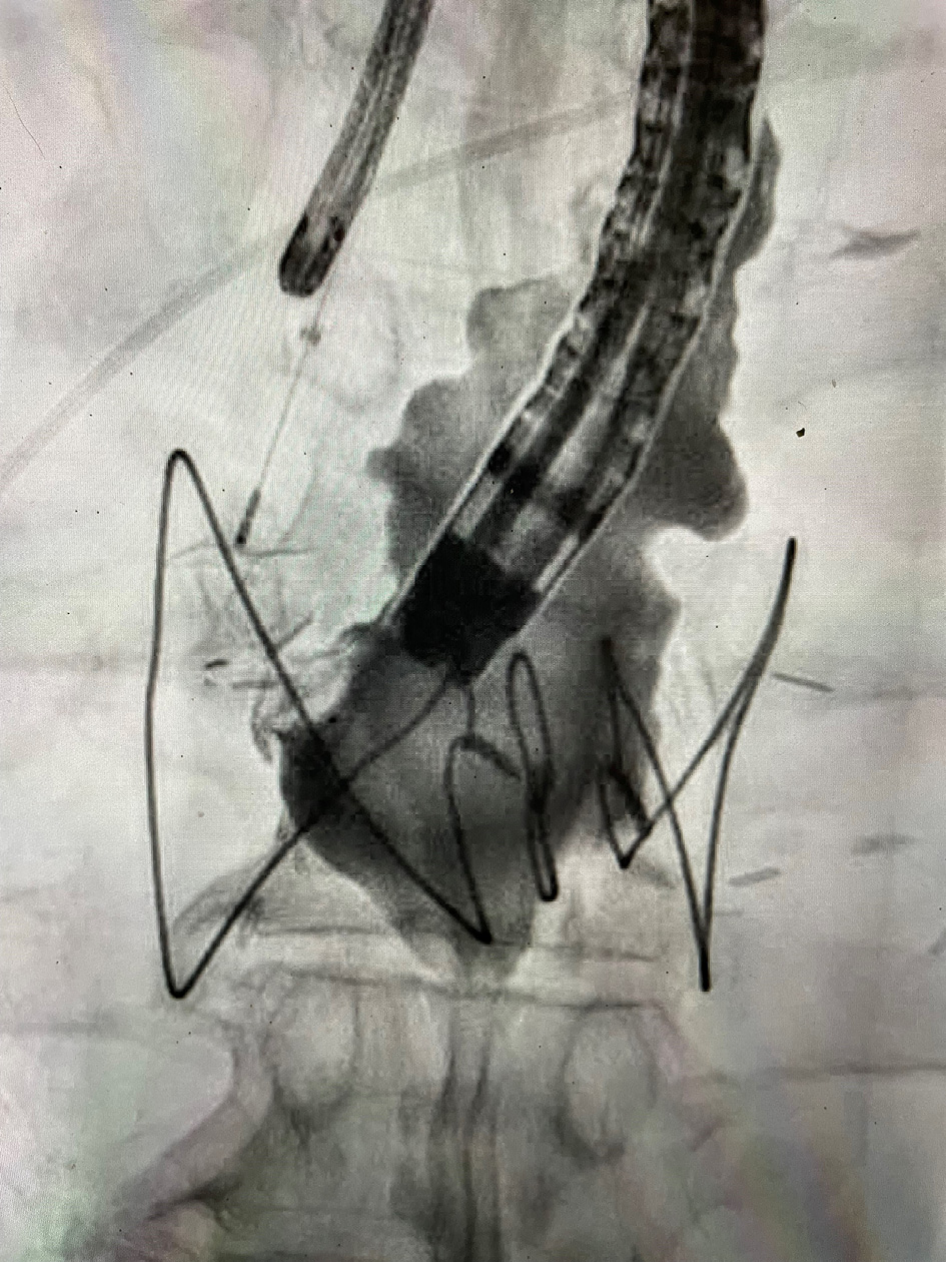
Intraluminal contrast material through the esophageal endoscope confirmed an adequate seal.

One year after this procedure, bronchoscopy showed the device well seated (Figure 3). During this year, several gastrointestinal complications developed, including an infected pancreatic pseudocyst. The patient was intubated for hypoxia, and a tracheostomy was performed. He remained critically ill, and in view of the poor likelihood of recovery, he received palliative care. The cause of death was unrelated to the bronchoesophageal fistula.

**Figure 3:**
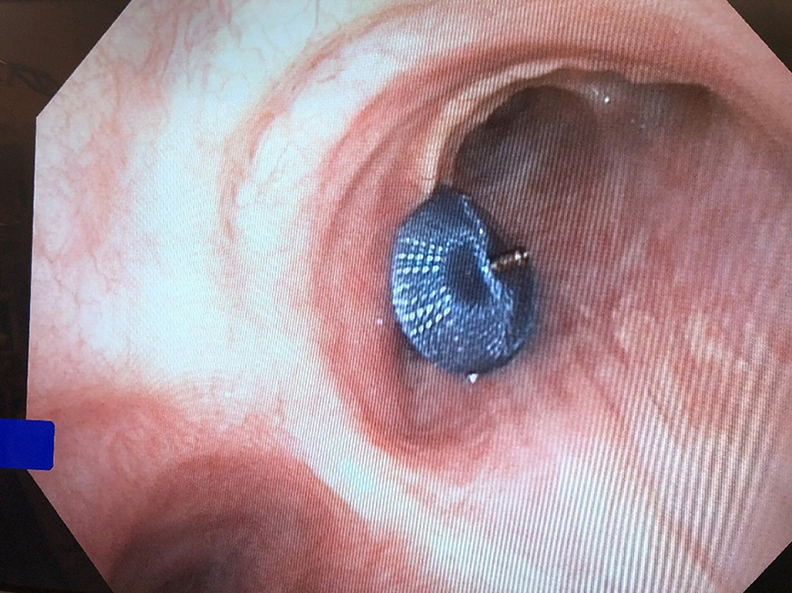
Bronchoscopy 1 year later. Device well seated without complications.

## Comment

Bronchoesophageal fistula is a rare lesion. Because of the magnitude of an open surgical approach, endoscopic or bronchoscopic stent placement is an attractive option.

Camagni and colleagues^1^ described a bilateral bronchial dehiscence with a right bronchoesophageal fistula after BLT, treated successfully with endoscopic double stenting. The off-label use of a cardiac septal occluder (CSO) device has been reported^2^, ^3^, ^4^, ^5^ to close fistulas between the airway and the esophagus. De Moura and coworkers^6^ reported 15 fistulas between the esophagus and the airway, the predominant cause of which was related to oncologic surgery; 77.27% of the patients had successful closure with a CSO device. Gómez López and colleagues^7^ described implantation of a CSO through a tracheostomy to close a bronchopleural fistula after a lobectomy in a patient who underwent BLT. The fistula was sealed and the last follow-up was performed 6 years later, with no evidence of complications.

In our case, the cause of the esophageal perforation is not clear. Possible mechanisms include devascularization of the esophageal wall during the preparation of the bronchial stump, passage of a stitch through the esophageal wall in performing the anastomosis, and erosion from a large hemoclip placed in a lymph node. It is possible that the transesophageal probe might have played a role.

In our opinion, an open surgical approach is the standard of care, but in some patients, like ours, with significant morbidity and mortality risk, bronchoscopic interventions are the next line of treatment. Airway stents are the most commonly used. In our patient, the stents did not fit well in the airways because of tapering bronchus intermedius and also because of the significant difference in diameter between the right main stem bronchus and bronchus intermedius. Custom-made 3-dimensional printed stents were not available at that time. Once the fistula became chronic and formed an epithelialized track, the bronchoscopic approach failed. In a patient with malnutrition and progressive loss of physical conditioning, placement of a CSO could represent a valuable option.

In conclusion, bronchoesophageal fistula after lung transplantation is a rare complication associated with significant morbidity and mortality. Because of the rarity of the lesion, management consensus is absent. Although an open surgical repair would usually be the first consideration, an endoscopic or bronchoscopic approach, given the magnitude of an open procedure, is attractive. In poor surgical candidates, closure of the fistula with a CSO device may be an option. Although this strategy was successful in this patient, because of the rarity of the lesion and likely publication bias, the real efficacy of this procedure remains unknown.
